# Autologous Deciduous Tooth-Derived Material for Alveolar Ridge Preservation: A Clinical and Histological Case Report

**DOI:** 10.1155/2020/2936878

**Published:** 2020-06-18

**Authors:** Elio Minetti, Silvio Taschieri, Stefano Corbella

**Affiliations:** ^1^Department of Biomedical, Surgical, and Dental Science, Università degli Studi di Milano, Milan, Italy; ^2^IRCCS Istituto Ortopedico Galeazzi, Milan, Italy; ^3^Federal State Autonomous Educational Institution of Higher Education I.M. Sechenov First Moscow State Medical University of the Ministry of Health of the Russian Federation (Sechenov University), 8-2 Trubetskaya St., 119991 Moscow, Russia

## Abstract

The management of the alveolar socket is fundamental to create conditions that would allow the positioning of dental implants in the same site, when required. A number of biomaterials were described in scientific literature to be used for alveolar socket preservation immediately after tooth extraction: autogenous grafts, allograft (of various origin), and synthetic products. Among the autogenous biomaterials, some authors proposed the use of autogenous dentin and/or enamel, retrieved from teeth, adequately managed, and purified. The present case report with two-year follow-up shows one case of alveolar socket preservation by using tooth graft material that was subsequently treated with one implant-supported rehabilitation in the same site. The paper presents clinical and histological outcomes and confirms the feasibility of adopting such autogenous biomaterial in standard procedures.

## 1. Introduction

The possibility of positioning dental implants in sites that underwent tooth extraction is strictly related on the amount of the available bone volume that results from bone healing and remodeling after tooth removal. Bone remodeling is a continuous, complex process that involves all the bone tissues in the organism and, obviously, could cause the resorption of alveolar bone when one tooth is no longer present [1]. In order to overcome the presence of insufficient available bone volume, a number of surgical procedures were described and validated by scientific literature aimed at increasing the bone volume [2].

As we know from the literature, after tooth extraction, the healing of the alveolar socket happens through the succession of many phases, beginning from the stabilization of the bone clot, the formation of fibrin, and, in the end, the recruitment of osteoblasts that will be responsible for new bone formation [3]. A number of biomaterials and technique were described for alveolar socket volume preservation, with many functions (osteoconduction, osteoinduction, or even stimulation) depending on the characteristics of each material [4]. MacBeth and colleagues in 2017 published one systematic review of the literature and reported that alveolar ridge preservation (ARP) may limit the necessity of further guided bone regeneration (GBR) procedures, reducing the bone changes during the healing period [4]. Such results confirmed what was found in previously published systematic reviews of the literature [5, 6]. However, all the authors concluded that ARP outcomes were extremely heterogeneous, and even though the techniques are effective, they cannot arrest the bone remodeling process and its reduction [7].

Considering studies about histomorphometric analysis, none of the commonly used biomaterials was found to be superior, in terms of new bone formation, as compared to the others [8].

In many clinical situations, the autogenous bone graft has been considered the most effective biomaterial [8, 9]. Human dentin and bone show many similarities in terms of mineralization. In fact, after demineralization, both the demineralized dentin matrix (DDM) and demineralized bone matrix (DBM) are predominantly composed of type I collagen (95%) and noncollagenous proteins (NCPs), and, among them, growth factors; for such reason, human DDM is a biological collagenous scaffold containing osteoinductive growth factors that provides an appropriate environment for stimulating new bone formation [10].

In 2015, Park et al. [11] suggested the use of the deciduous tooth as source of graft materials. They made an in vivo study, and the conclusion was that the deciduous teeth had structural and physicochemical characteristics suitable for grafting with appropriate demineralization.

In 2018, Bono et al. [12] described that deciduous teeth could be used as grafting materials in bone augmentation procedures. They also shed light on the effects of demineralization on deciduous teeth material, evaluating whether or not collagen and BMP-2 protein contents were preserved after the chemical treatment. Moreover, they evaluated in vitro the response of osteoblastic cells to exogenous BMP-2 stimulation (at different protein concentrations) with the aim of identifying the minimum BMP-2 concentration able to induce the expression of alkaline phosphatase, the early marker of the osteoblastic phenotype.

The aim of the present report is to present a case of ARP using autogenous deciduous tooth material, presenting both clinical and histologic results.

## 2. Case Presentation

The subject of the case report was one 26-year-old women, nonsmoker, ASA-1 (following the classification proposed by the American Society of Anesthesiologists), and able to understand the information given about the treatment.

**Figure 1:**
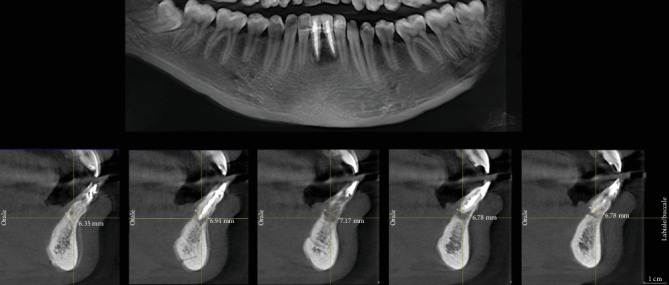
Radiographs of preoperative situation.

The patient referred pain and mobility in the area of 3.1 and 4.1 (mandibular central incisors). The radiographic (by periapical radiograph and CBCT) and clinical examination revealed that the teeth (both) underwent nonsurgical endodontic treatment that was unsuccessful. It was evident the presence of a large bone loss area in the periapical region with partial preservation of the buccal bone. The teeth were both affected by mobility (more than 2 mm), suppuration, and spontaneous pain (Figure 1).

For this particular case, the treatment alternatives were limited. The operator proposed the removal of both teeth, alveolar ridge preservation, and, after healing, the placement of two dental implants to support one fixed prosthesis. The patient approved the proposed treatment protocol, accepting to use her own deciduous teeth (stored by the patient in plastic box for at least 15 years) as bone grafting material. The decision of using deciduous teeth materials was due to the impossibility of using the extracted teeth for the same purposes. In fact, the amount of potentially available material, after removing the endodontic fillings, would not be sufficient in such clinical situation. The patient signed an informed consent form before surgery.

Before surgery, as standard protocol, the patient received professional oral hygiene with supragingival scaling.

After local anesthesia performed with articaine 4% and epinephrine 1 : 100.000 in the 3.1-4.1 position, the extraction of affected teeth was performed without flap elevation, preserving the buccal bone plate. A piezoelectric device was used to debride the alveolar socket, removing infected tissues [13].

In order to use deciduous teeth as bone substitute material, the following procedure was adopted: (1) the teeth were adequately selected avoiding teeth with caries and cleaned, (2) the teeth were cut in small portions, and (3) they were grinded, demineralized, and sterilized using a specific device (Tooth Transformer®, Milan, Italy), following a standard and automatic procedure.

The so-obtained biomaterial, weighting approximately 1.5 grams (made of both dentin and enamel, in granules with diameter ranging between 0.4 and 0.8 mm), was placed and compacted in the alveolar sockets and the soft tissues were sutured with bioresorbable material (Vicryl 5-0, Ethicon Inc., Johnson & Johnson, Bridgewater, USA).

Standard postoperative instructions were provided, prescribing the use of 15 mL 0.2% chlorhexidine mouthwashes twice a day for seven days and nonsteroidal anti-inflammatory drug (ibuprofen 600 mg twice a day for three days).

**Figure 2:**
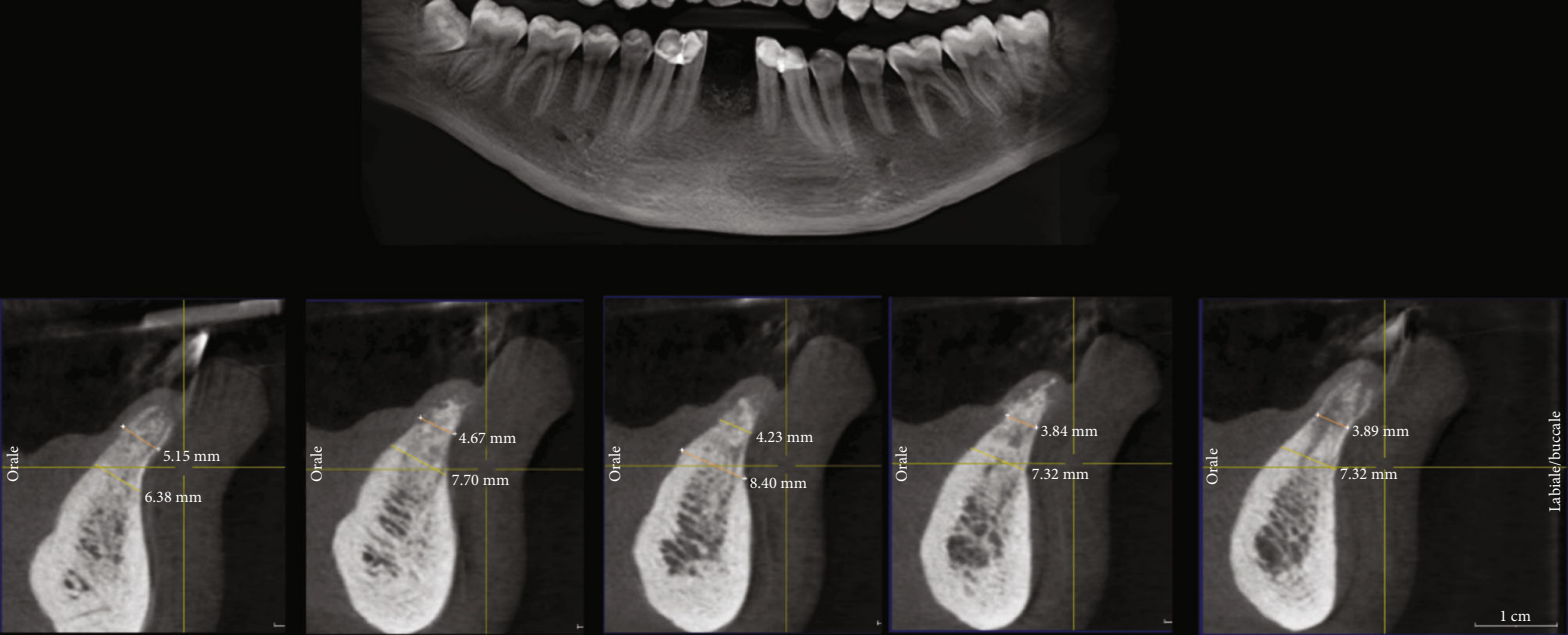
Section of CBCT scan, taken after 5 months, showing bone healing after grafting.

**Figure 3:**
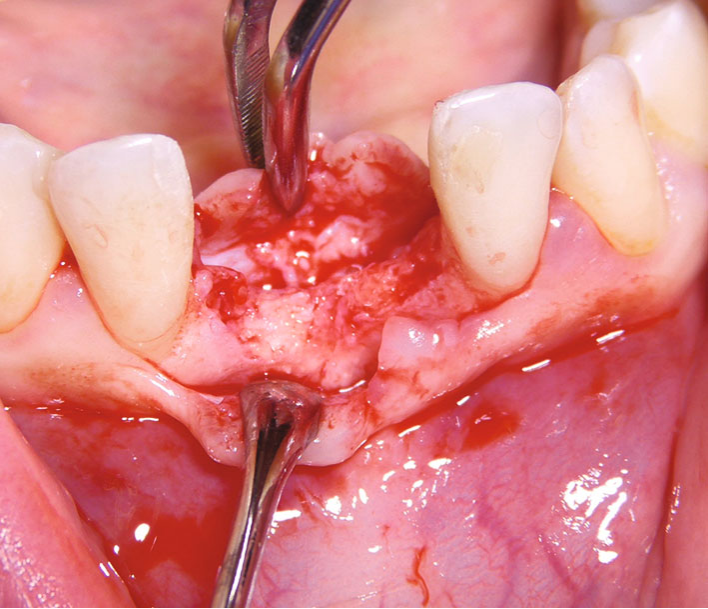
Clinical image showing a sufficient amount of bone volume.

**Figure 4:**
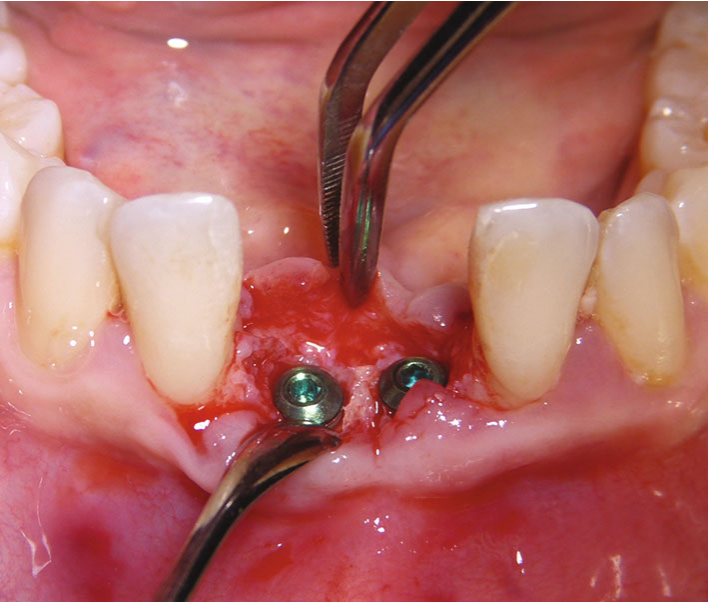
Clinical image showing the situation after placement of the two dental implants.

After five months from the ARP intervention, we performed a CBCT scan to evaluate the healing of the bone and to plan dental implant placement (Figure 2). The radiographic investigation revealed the presence of sufficient available bone to place two 3.5 × 13 mm implants (Visio One ®, CEA Medical Sa, Geneva, Switzerland). After local anesthesia, a full-thickness mucoperiosteal flap was raised in the 3.1-4.1 region, and it was possible to confirm the presence of available bone volume and quality to place two dental implants in that region, without needing further procedures for bone augmentation (Figure 3). While preparing the implant site, using a 3 mm trephine bur, a biopsy of the bone tissues was taken with the aim of analyzing the characteristics of the healed bone in the region of autogenous grafting (Figure 4). The surgical procedure ended after implant placement by suturing the repositioned flap. Healing was uneventful. Second stage surgery was performed after three months, and the metal-ceramic prosthesis was delivered after a total of nine months from the ARP procedure.

**Figure 5:**
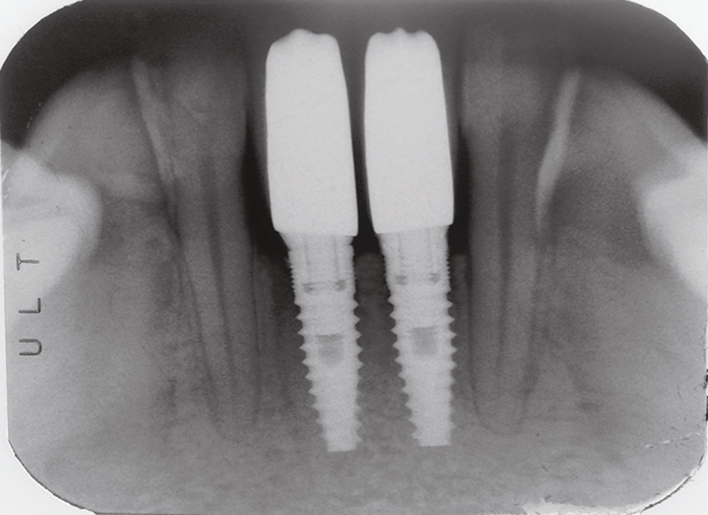
Periapical radiograph taken 2 years after prosthetic loading.

The clinical and radiographic follow-up revealed, after two years from loading, the absence of bone resorption process and stability of soft tissues (Figure 5).

### 2.1. Histologic Analysis

The histologic analysis was performed in the laboratory of the Biomaterial Clinical and Histological Research Association in Pescara, Italy.

The samples were dehydrated by a series of solutions with increasing alcohol concentration, up to pure alcohol, and then infiltrated into a methacrylic resin. After light curing of the resin, the sample was processed to obtain nondecalcified sections due to wear, using a disk abrasion system (LS2-Remet, Bologna) and a diamond disk cutting system (Micromet-Remet, Bologna). In the first phase, the inclusion in resin was abraded to eliminate the resin component that covered the sample; the area of the biopsy to be observed was thus brought to the surface. Then, the surface was glued to a showcase with cyanoacrylate-based adhesives. Subsequently, cutting with a high speed and cooling diamond blade was performed. In this way, a slide with a sample of about 200 microns thick was obtained which must be thinned by abrasion. With low abrasive paper, the sample was then abraded on the lapping machine with thickness control that allowed to progressively reduce the sample thickness up to about 40-50 microns. At this point, the slide was polished with polishing papers and colored with basic fuchsin and blue toluidine for the final observation in light and polarized light microscopy.

For histomorphometric measurements, the histological images obtained from the transmitted light microscope were digitized through a digital camera and analyzed by means of the image analysis software IAS 2000.

For each sample we calculated the following: BV% is the percentage of residual bone volume with exclusion of medullary tissues; Graft% is the percentage of the remaining graft, excluding the bone and marrow; and VB% is the percentage of vital bone with exclusion of the medulla and residual graft.

**Figure 6:**
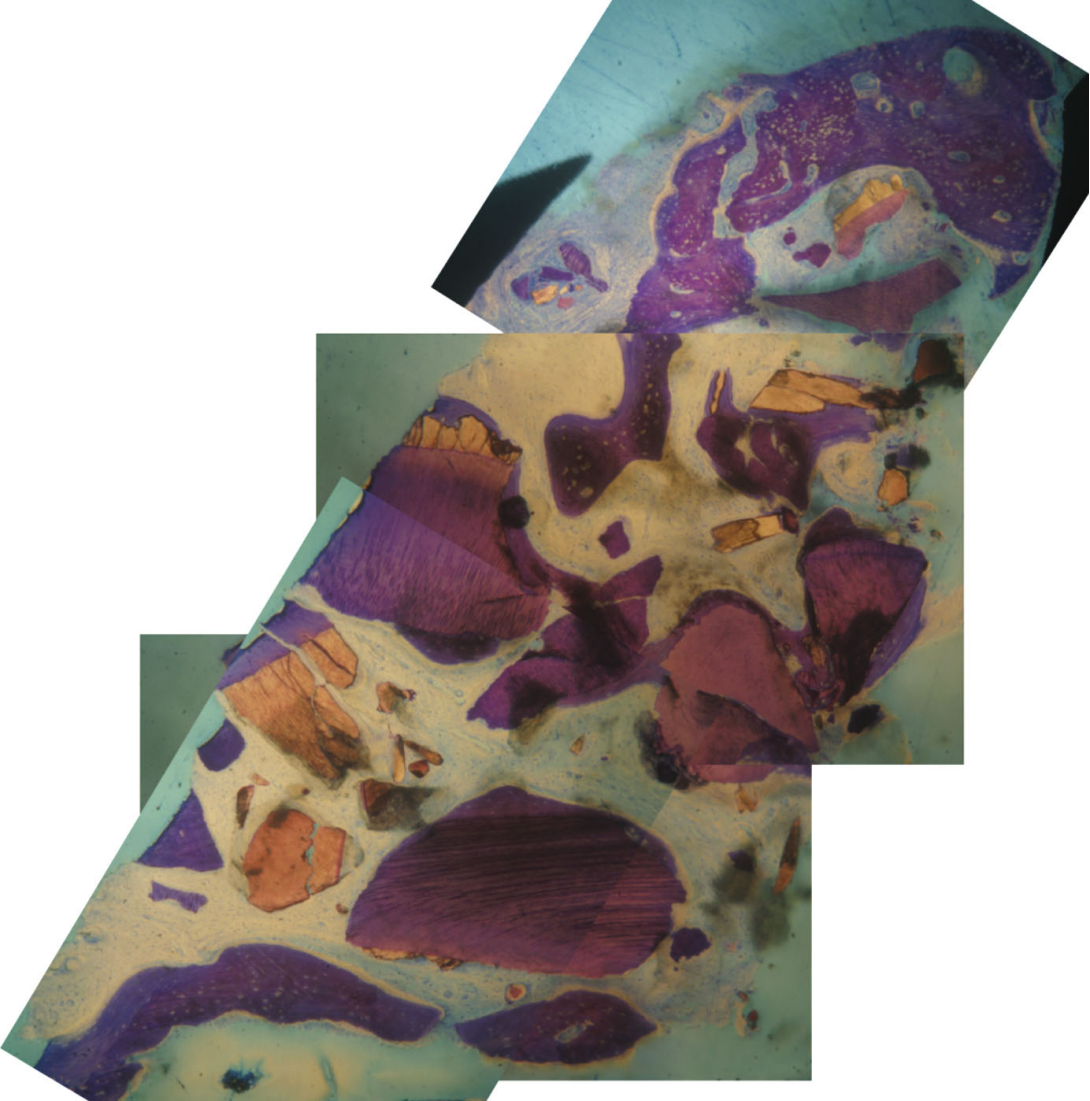
Section used for histologic analysis. In dark violet, the tooth-derived material; in violet, the bone, with Havers' canals.

The histologic analysis (Figure 6) revealed 47.22% of BV, 18.68% of graft volume, and 28.55% of VB, indicating a substantial integration of the biomaterial used without signs of inflammation.

## 3. Discussion

The present case report demonstrated that deciduous teeth could be used as a source of bone substitute material, with good results in terms of histologic integration of the biomaterials and of clinical outcomes, thus allowing an effective ARP procedure.

Healing of the extraction socket has been widely studied by a number of published papers of high quality [3, 14]. One of the most important factors in determining the extent of the bone resorption process is represented by the presence of the buccal bone wall and by its width [14]. The so-called ARP techniques were proposed in order to maintain the bone volume even after tooth extraction and to reduce the need of performing complex and invasive bone augmentation procedures to place dental implants [4].

Over the years, a number of bone substitute materials were described in alveolar socket preservation procedures, but none of them demonstrated significantly better outcomes from the histomorphometric analysis [8].

For this case report, we decided to use such autologous material for several reasons, which represents the main advantages of the presented technique. First is the fact that we have great availability in terms of volume, then the use of completely autogenous material would have been beneficial in order to lower the possibility of adverse immune reactions, the patient refuses receiving biomaterials of animal origin, and finally, the use of the deciduous tooth was completely free of any biological or economic cost. Moreover, on the basis of the results of the research performed by Schmidt-Schultz and Schultz in 2005, we assumed that the characteristics of the teeth were not changed over the storing period, maintaining both the mineral and the nonmineral composition [15]. Therefore, the decision of using deciduous teeth was supported by the fact that they have structural and chemical characteristics that are suitable for grafting, having less enamel than permanent teeth, and, thus, an increased osteoinduction and resorption rate [11].

There are several papers about the use of tooth materials (dentin and enamel) as bone substitutes, acknowledging the advantage of being available after tooth extraction, thus avoiding other surgical procedures to be grafted (as it happens for the block graft from the mandibular ramus, for example) [10, 16, 17].

In 2018, one systematic review of the literature was published by Gual-Vaques et al., with the aim of evaluating autogenous teeth used as bone grafting material before implant placement [10]. The authors examined the existing literature of about 6 papers for a total of 182 dental implants. On the basis of the available data and admitting that the number of papers was insufficient for conclusive considerations, the authors stated that the use of such biomaterial was safe and effective for allowing implant placement in sites requiring bone augmentation [10].

The positive outcomes of the case we treated found support by other studies describing similar cases, even though not using deciduous teeth. The pilot study published by Del Canto-Diaz et al. in 2019 reported the results obtained using autogenous tooth material in nine patients. The outcomes they obtained were promising, since bone volume changes were significantly lower in the test group than in sites left to heal spontaneously [18]. Similar results were found also in more demanding bone regeneration procedures, such as the treatment of furcation defects [19].

To our knowledge, this is the first case described in the literature of regeneration performed in an adult using an autologous deciduous tooth. The present case report validity is limited by the study design itself. More studies (randomized controlled clinical trials) with a larger sample size are needed to confirm the results obtained in a significant number of pilot studies.
